# Supplementary material for: A set of multi-entry identification keys to African frugivorous flies (Diptera, Tephritidae)
Source: Zookeys. 2014 Jul 24;(428):97–108. doi: 10.3897/zookeys.428.7366 (PMC4143993; doi:10.3897/zookeys.428.7366)
Supplement: Supplementary material 6 — Key to Ceratitis [file zookeys-428-097-s006.zip › SF6_ZooKeys_key to Ceratitis/key/SF6_ZooKeys_key to Ceratitis/Media/Html/Ceratitis acicularis .htm]

Ceratitis (Pterandrus) acicularis (Munro)


***Ceratitis*** **(*Pterandrus*) *acicularis*
(Munro)**

*Pterandrus acicularis* Munro, 1969: 420.

Body length: 5.09 (4.80-5.30) mm; wing length: 5.68 (5.25-6.15) mm.

Male

Head:
Antenna yellow. First flagellomere 2-3 times as long as pedicel. Arista with
moderately long rays; ventral rays somewhat shorter and sparser than dorsal
rays, especially basally. Frons yellow; with short scattered setulae largely of
same color as frons. Frontal setae well developed, occasionally less so. Face
yellowish white. Genal seta and setulae dark, well developed.

Thorax:
Postpronotal lobe white, without spot. Scutal pattern, ground color pale gray,
with streaks and darker markings but without distinct spots or clearly defined
stripes, except prescutellar white markings, separate but with pale area in
between. Scapular setae dark. Scutellum grayish white, basally without spots,
apically with three separate black spots, extending to basal half. Anepisternum
completely pale, ventral margin narrowly dark brown; setulae pale except few
dark setulae usually present along ventral margin.

Legs: Yellow
except where otherwise noted; setation typical for subgenus, mainly dark.
Foreleg: femur anteriorly mainly brownish, only basal and ventral margin
yellow, posteriorly largely brown, no distinct bush, only dispersed setulae;
ventral setae black. Midleg: femur anteriorly largely brown, distal end pale;
ventrally with dark dispersed setulae, distally somewhat denser but without
distinct feathering; tibia broadened; largely brown, with black feathering dorsally
along distal 0.9 and ventrally along distal 0.8, anterior part covered by short
dark setulae. Hindleg: femur brown on basal 0.8; at apical 0.25 with longer
setulae dorsally and ventrally.

Wing:
Bands brownish. Interruption between marginal and discal bands near vein R_1_
clear and complete; discal band sometimes partly interrupted in discal cell;
cubital band free; medial band absent; crossvein R-M opposite middle of discal
cell. Apex of vein R_1_ distal to level of crossvein R-M. Crossvein
DM-Cu straight.

Abdomen: Mostly
Yellow. Tergites 2 and 4 largely pale gray. Tergite 3 with brownish black
transverse band along posterior half. Tergite 5 along anterior margin with
isolated brownish patches, posterior margin narrowly black. Male epandrium in
lateral view with posterior lobe of lateral surstylus straight and elongate.

Female

As
male except following characters: Legs without feathering, yellow, except
femora yellowish orange; forefemur posteroventrally with dark pilosity. Discal
band not interrupted. Tergite 5 largely pale yellow across posterior half.
Oviscape as long as, or longer than, preabdomen. Aculeus at least eight times
as long as wide; tip with distinct apical indentation and lateral margin
slightly sinuous.

(Description
after De Meyer & Freidberg, 2006)
